# Supplementary figures and images for: Origins of East Caucasus Gene Pool: Contributions of Autochthonous Bronze Age Populations and Migrations from West Asia Estimated from Y-Chromosome Data
Source: Genes (Basel). 2023 Sep 9;14(9):1780. doi: 10.3390/genes14091780 (PMC10530682; doi:10.3390/genes14091780)

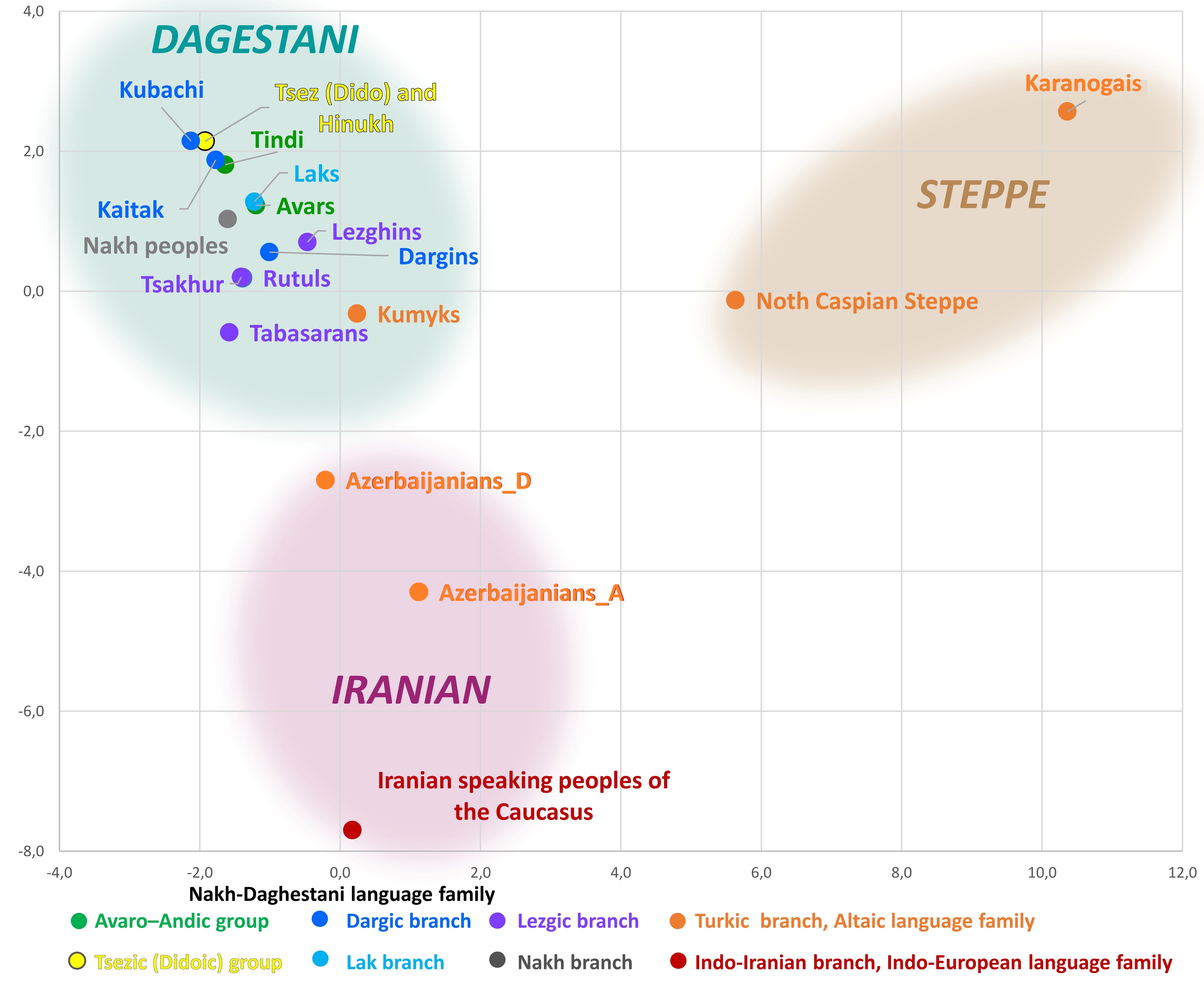

Supplement: Supplementary file 1 [file genes-14-01780-s001.zip › Figure S1.jpg]

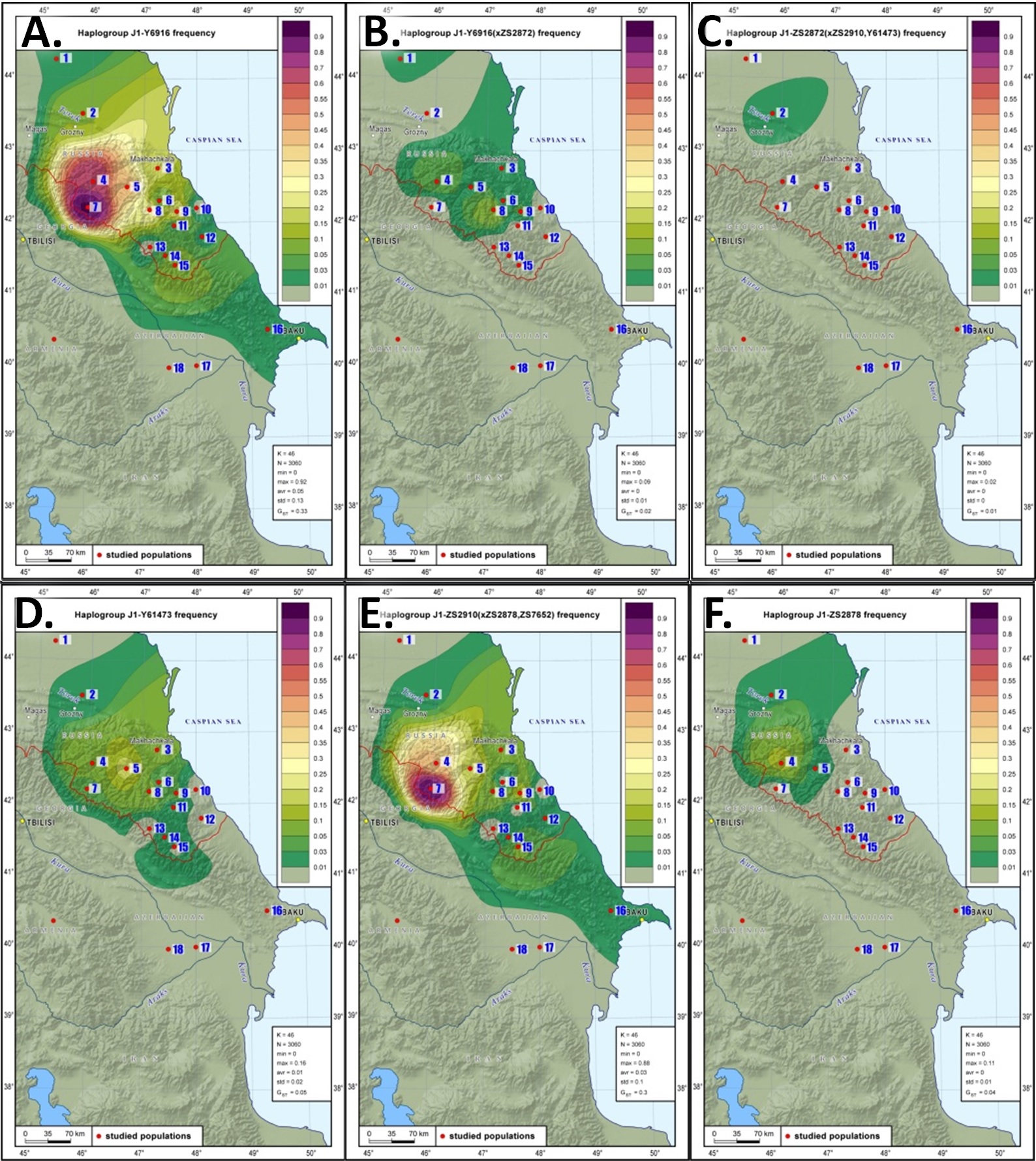

Supplement: Supplementary file 1 [file genes-14-01780-s001.zip › Figure S2.jpg]
